# Music-Based Cognitive Training for Adults with Major Depressive Disorder and Suicide Risk: A Pilot Study

**DOI:** 10.3390/jcm14030757

**Published:** 2025-01-24

**Authors:** Melissa Tan, Steffi Friyia, Corene Hurt-Thaut, Sakina J. Rizvi, Michael H. Thaut

**Affiliations:** 1 Music and Health Sciences Research Collaboratory, Faculty of Music, University of Toronto, Toronto, ON M5S 2C5, Canada; 2 ASR Suicide and Depression Studies Unit, St. Michael’s Hospital, Toronto, ON M5B 1M4, Canada; 3 Department of Psychiatry, University of Toronto, Toronto, ON M5T 1R8, Canada; 4 Faculty of Medicine, University of Toronto, Toronto, ON M5S 3K3, Canada

**Keywords:** suicide risk, depression, cognition, music-based intervention, neurologic music therapy

## Abstract

**Background/Objectives**: Cognitive challenges in attention and executive function worsen over time in individuals with major depressive disorder (MDD) and suicidal risk. These difficulties persist beyond acute episodes, with limited targeted treatments available. Neurologic music therapy (NMT) is effective for cognitive rehabilitation in brain injuries and developmental disabilities, suggesting potential benefits for adults with MDD and suicide risk. This pilot study evaluated the feasibility, acceptability, and preliminary effectiveness of short-term NMT on cognitive function in adults with MDD. **Methods**: Adults aged 18+ with MDD and suicidal ideations participated in an 8-week single-arm open label study with 45-min individual in-person NMT sessions using musical attention control training (MACT) and musical executive function training (MEFT). Participants provided feedback on feasibility and acceptability, and pre- and post-intervention assessments included neurocognitive tasks and questionnaires on suicidal ideation, depressive symptoms, and quality of life. **Results**: A total of 18 individuals enrolled, and 10 participants completed the study protocol. Of the participants, 100% were satisfied with their experience with NMT, with 100% noting improvements in attention and 80% in executive function. Participants experienced some improvements in short-term memory (Digit Span Forward Test), cognitive flexibility (Trail Making Test B), and inhibitory control (Stroop Task). Significant reduction in suicidal ideation intensity (Beck Suicidal Scale of Ideation) was observed, as well as significant improvements in quality of life. **Conclusions**: This is the first study using NMT to demonstrate feasibility, acceptability, and effectiveness with respect to cognitive function in adults with MDD and suicide risk, providing preliminary data for future randomized controlled trials.

## 1. Introduction

Suicide is a global public health concern, with more than 720,000 people dying annually. It is the fourth leading cause of death for individuals aged 15–29 [1]. Individuals diagnosed with major depressive disorder (MDD) are at higher risk of suicidal behavior [2]. The prevalence of suicide and increased risk among individuals with MDD underscore the urgent need for feasible and acceptable interventions that can effectively address the needs of those at risk.

A critical area where support is needed is cognitive function. Cognitive challenges such as rigidity, impaired problem solving, and dichotomous thinking are common in individuals with suicidal ideation [3]. Research shows that attention, working memory, and inhibition are particularly impaired in those with MDD and a history of suicide attempts, compared to non-attempters and healthy controls [4]. Cognitive dysfunction appears to worsen over time, persisting even when depressive episodes are in remission [5]. While antidepressant pharmacotherapy may have some impact on cognitive function, deficits in concentration and decision making often persist [6,7,8].

Cognitive functioning may show more specific deficits in individuals with past suicide attempts, such as difficulties with interference control [9], whereas individuals with MDD with no attempt tend to exhibit more general deficits, including challenges with attention, memory, executive functioning, processing speed, and verbal fluency [10]. In individuals with MDD and past suicide attempts, selective attention as well as general attention and memory are impaired compared to non-attempters and healthy controls [9,11]. These impairments can severely impact an individual’s daily and occupational functioning [10]. This underscores the importance of targeted interventions to enhance cognitive functioning with the possibility of alleviating depressive symptoms and suicide risk [9,10,11].

Music-based interventions offer a promising avenue to alleviate cognitive impairments in individuals with MDD and suicide risk. The application of music-based interventions in clinical settings is currently being used to address cognitive function and psychosocial needs in other psychiatric populations that also face similar cognitive dysfunctions due to post-traumatic stress disorder and substance abuse [12,13]. Neurologic music therapy (NMT) has been proven to be effective for cognitive function, addressing areas in attention and perception training, memory training, executive function training, and psychosocial behavior training [14]. Musical attention control training (MACT) and musical executive function training (MEFT) are two techniques that will be explored in the present study.

MACT has been used with various populations, including individuals with ADHD, autism, acquired and traumatic brain injuries, and dementia [14]. A study involving adolescents in secured residential youth care with attention difficulties showed significant improvements in focused, sustained, and alternating attention following participation in MACT, compared to a non-standardized music therapy intervention [15]. Similarly, a pilot study in a forensic psychiatric hospital with patients experiencing psychotic symptoms found that those receiving six weeks of weekly 30-min MACT sessions, in addition to treatment as usual, demonstrated significant improvements in sustained, selective, and alternating attention compared to a control group [16].

MEFT has shown promise for individuals with acquired brain injuries, attention deficit disorders, and neurologic conditions like Parkinson’s disease and multiple sclerosis [17]. Participants with traumatic brain injuries who engaged in four 30-min NMT sessions demonstrated significant improvements in cognitive flexibility [18]. Similarly, individuals with traumatic brain injuries who participated in weekly NMT and psychotherapy sessions experienced significant improvements in visual attention, verbal learning, memory, planning, and mental flexibility [19].

There is an urgent need for solutions to improve cognitive functioning in adults with mental health conditions. This pilot study explores the feasibility and acceptability of neurologic music therapy as an adjunctive intervention to support individuals with MDD and suicide risk, as well as assessing the effectiveness on attention and executive function skills.

## 2. Materials and Methods

### 2.1. Participant Recruitment

Participants were recruited between July 2023 and April 2024 through three main sources: the Department of Psychiatry at St. Michael’s Hospital (Toronto, Ontario), an existing database of individuals who had given consent to be contacted for future studies, and advertisements placed in the community and on Facebook.

All participants met the DSM-V diagnosis for a current major depressive episode (MDE), as confirmed by the mini-international neuropsychiatric interview (MINI), and were experiencing suicidal ideation in the past week (Beck Scale for Suicide Ideation ≥ 10). All participants were required to have more than 12 sessions of psychotherapy for their current depressive episode and, if on medication, were on a stable regimen for at least four weeks; they were required to have not participated in music therapy prior to the study, to have had no private music lessons for a period of at least one year prior to participation, and no presence of cognitive impairment based on self-report and clinician judgment. Participants were excluded if they had impaired or corrected hearing, if there was a presence of active psychosis, and if there was the presence of mood and suicidal symptom severity requiring immediate treatment. Participants were withdrawn if they initiated new psychotropic medication or new psychotherapy treatment during the study.

Participants provided written informed consent prior to participation. The study was approved by the Unity Health Toronto Research Ethics Board (REB#22-280) and was also registered at ClinicalTrials.gov (NCT05694156).

### 2.2. Study Design

**Figure 1:**
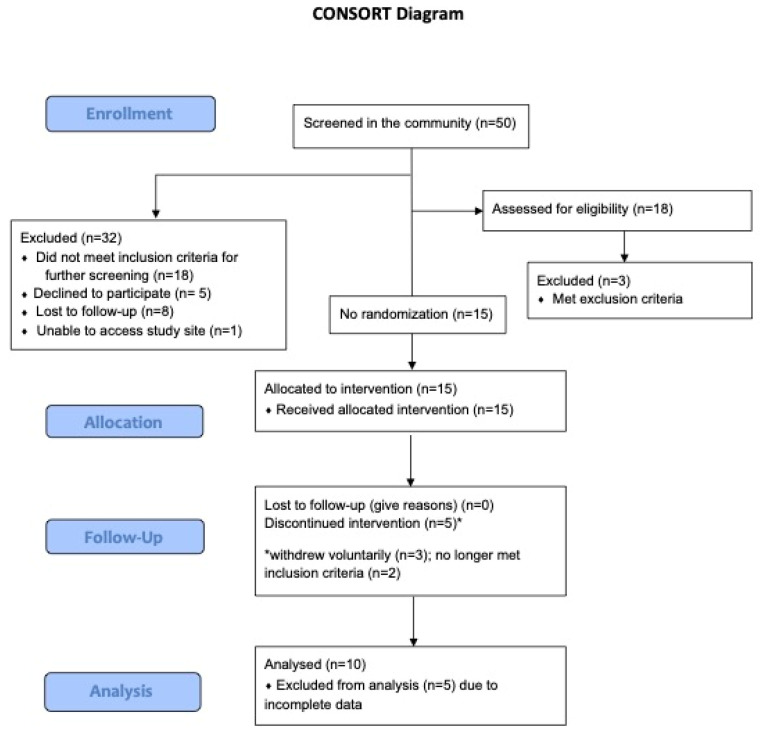
CONSORT study diagram for a single-arm open-label pilot music-based cognitive intervention study involving adults with MDD and suicide risk.

**Figure 2:**
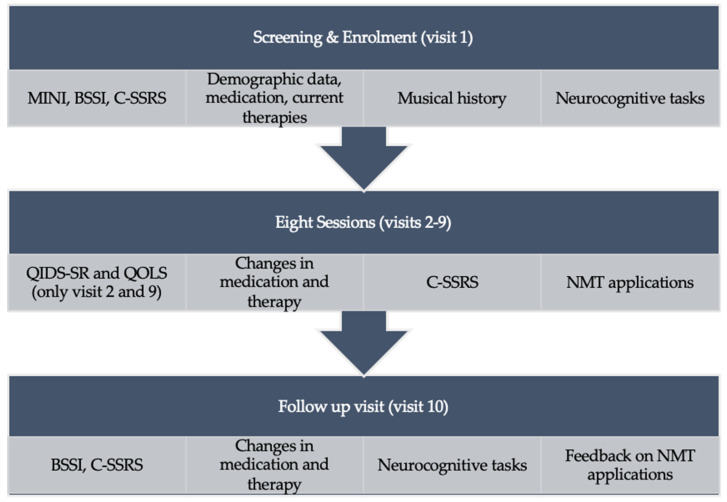
Study design. Abbreviations in order of appearance: mini-international neuropsychiatric interview (MINI); Beck Scale for Suicide Ideation (BSSI); Columbia Suicide Severity Rating Scale (C-SSRS); Quick Inventory of Depressive Symptomatology—Self-Report (QIDS-SR); Quality-of-Life Scale (QOLDS); neurologic music therapy (NMT).

This study utilized a single-arm open label design (see Figure 1). Upon the provision of written informed consent, participants had ten in-person visits: a screening visit to determine eligibility and complete neurocognitive assessments, eight individual music-based cognitive training sessions, and one follow-up visit where they were reassessed (see Figure 2).

#### 2.2.1. Study Outcome Measures

The feasibility and acceptability of music-based cognitive training sessions were measured by an in-house survey that included both Likert scale questions and open-ended responses (i.e., accessibility, experience, usefulness, relevance, and suggestions for improvement).The Likert scale, rated on a 5-point system, captured key insights on overall satisfaction, ease of incorporating sessions into their weekly schedules, perceptions of improvements in attention and executive function, and the overall helpfulness of the experience.

To assess cognitive function, the following computerized neurocognitive tasks were used: Digit Span Forward and Backward for short-term memory and working memory; Trail Making A and B Tests for processing speed, cognitive flexibility, and set shifting; the Stroop Task for ability to inhibit cognitive interference; and the Go/No-Go Test for inhibitory control. Inquisit Lab was used to administer the Digit Span, Trail Making Tests, and the Stroop Task. E-Prime was used to administer the Go/No-Go Test.

Suicidal ideation severity was assessed using the Beck Scale for Suicide Ideation (BSSI) and the Columbia Suicide Severity Rating Scale (C-SSRS). Depressive symptoms were measured by the Quick Inventory of Depressive Symptomatology—Self-Report (QIDS-SR). Quality of life was assessed using the Quality-of-Life Scale (QOLS).

#### 2.2.2. Procedures

A screening visit (visit 1) was undertaken to conduct a structured diagnostic interview for DSM-V disorders (MINI) and suicidal ideation severity. Demographic data, current medication use, current therapies, and musical history were also collected. The participants completed computerized neurocognitive tasks to assess cognitive functions (i.e., memory, processing speed, cognitive flexibility, and inhibitory control).

Participants were then scheduled for eight in-person individual music-based cognitive training sessions using MACT and MEFT (visits 2–9), which were designed to function as adjunctive therapy. Each session was 45 min, delivered by a licensed psychotherapist (MT) who was also a certified neurologic music therapy fellow (NMT-F) and a certified music therapist (MTA). During the first and last music-based cognitive training sessions (visit 2 and visit 9, respectively), participants completed questionnaires to assess depressive symptoms and quality of life.

Before each of the sessions, and during the follow-up visit (visit 10), the NMT-F or a member of the research staff asked about changes in medication, involvement in psychotherapy, and administered the C-SSRS. After each of the eight music-based cognitive training sessions, the NMT-F completed a post-session form. This documented the music-based tasks completed during the session, the success of completion, and any additional notes of what occurred during the study visit.

The final visit (visit 10) was conducted by a trained research coordinator, who administered the BSSI, neurocognitive tasks, and collected information about changes in psychotherapy participation and medication. Participants completed an in-house survey to obtain feedback on their experience. The follow-up visit was held after the last music-based cognitive training session (visit 9), either on the same day or the following day.

### 2.3. Music-Based Cognitive Training Protocol

**Figure 3:**
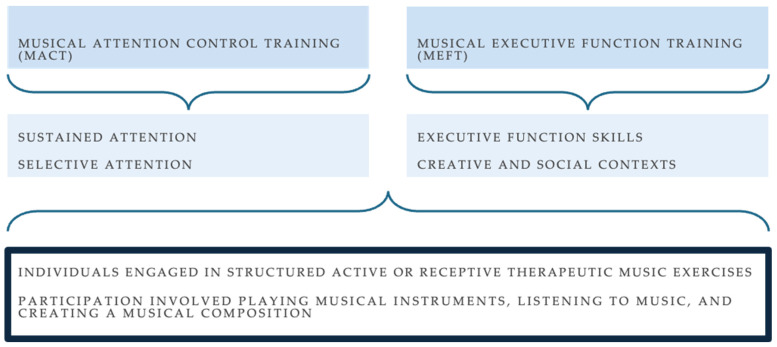
Music-based cognitive intervention schematic. Participants engaged in two NMT applications for cognitive training: MACT and MEFT.

Participants did not have to have prior musical knowledge to participate in music-based cognitive training. All exercises were structured using cognitive NMT applications, specifically musical attention control training (MACT) and musical executive function training (MEFT) (see Figure 3).

#### 2.3.1. Musical Attention Control Training (MACT)

The exercise protocols derived from MACT consisted of interactive music improvisation exercises between the participant and therapist on various instruments, including the keyboard and various percussion instruments. MACT exercises used structured or receptive therapeutic musical exercises where musical elements cued different responses to practice sustained and selective attention functions. The participant was provided target cues to which they had to respond in predetermined ways. Musical cues included specific melodies, harmonic chords, or instrument changes. Verbal cues included short instructions to prompt a particular musical response. Participant responses to the cues were musical adjustments in playing, i.e., “stop–go”, “change in tempo”, “change in instrument”, “change in pitch register”, and “change in loudness”.

Selective and sustained auditory attention training required participants to listen to all the musical events and identify a specific target cue among them, responding in a pre-determined way. These exercises emphasized on improving the flexibility and adaptability of the auditory attention system by offering a variety of cues and corresponding responses.

#### 2.3.2. Musical Executive Function Training (MEFT)

The exercise protocols derived from MEFT used improvisation and composition exercises to stimulate executive function skills, including organization, problem solving, decision making, reasoning, comprehension, and inhibition, within a social context.

Participants engaged with MEFT exercises that train inhibition where the participant was asked to clap or play an instrument (i.e., handheld percussion) and rest during specific times within a rhythmic pattern (e.g., the participant is instructed to play to a 4-beat rhythm and is instructed to rest on beat 3). The therapist provided verbal cues to encourage the desired musical response. The exercise integrated the opportunity for participants to become self-aware of when they had the desire to respond musically, while simultaneously exercising their ability to inhibit specific musical responses.

Participants also took part in structured musical composition exercises, guided step-by-step through an executive dialogue. This process involved a series of questions and responses to help with decision making, problem solving, reasoning, comprehension, organization, initiation, inhibition, evaluation, analysis, and creativity. The dialogue began with closed-choice questions (e.g., the therapist played different musical options for the participant to choose from) and gradually progressed to open-ended questions, allowing participants to explore musical ideas independently. Compositions were created and performed using percussion and keyboard instruments, integrating the real-time development of both the final product and the process while combining emotional and cognitive components at each stage.

### 2.4. Statistical Analysis

The feasibility of the pilot study was examined through the evaluation of rates of recruitment, data completion, retention, and feedback survey data. Qualitative content analysis (QCA) was implemented to analyze open-ended responses provided in the feedback survey. Two researchers independently read and coded responses into themes and categories using an inductive approach. Summaries were then compared to establish and finalize themes and categories. Common patterns, insights, and example quotations were then identified, providing a fuller understanding of the participants’ experiences.

To assess cognitive and clinical outcomes, paired samples *t*-tests were used to compare results before and after the music-based intervention at baseline and follow-up (visits 1 and 10, respectively). The Wilcoxon signed-rank test was used when normality was not met. Effect sizes were calculated to assess the magnitude of the music-based intervention’s impact. Cohen’s *d* was used to measure the effect size for the paired samples *t*-test, while the effect size (*r*) was calculated for the Wilcoxon signed-rank test.

All statistical analyses were conducted in R version 4.4.1.

## 3. Results

### 3.1. Participant Description

Of the 50 individuals screened for eligibility, 18 participants were enrolled to the study (see Figure 1). Ten participants completed the study protocol described. Study discontinuations were due to initiating new medication and/or psychotherapy (n = 2), time commitments (n = 2), difficulties in completing the weekly C-SSRS assessment (n = 1) and meeting exclusion criteria at the time of enrollment (n = 3). Two participants withdrew after three sessions, two participants withdrew after one session, and one participant withdrew after six sessions. On average, participants completed the eight session protocol over 10 weeks (rage 8–22 weeks). Participant baseline characteristics are described in Table 1.

### 3.2. Neurocognitive Tasks Outcomes

**Figure 4:**
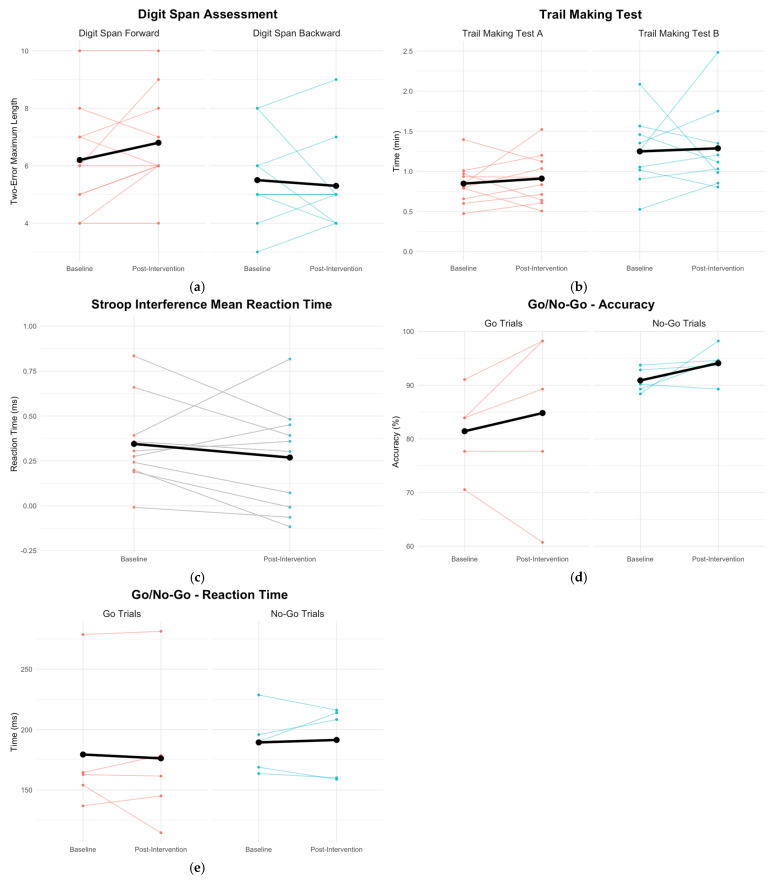
Line graphs represent effects of music-based cognitive intervention at baseline and post-intervention for neurocognitive tasks. Raw scores of each participant and mean scores (black line) are represented: (**a**) Digit Span assessment forward and backward scores of two-error maximum length; (**b**) Trail Making Test A and B (with outlier data) times in minutes; (**c**) Stroop interference mean reaction time in milliseconds; (**d**) Go/No-Go correct responses; (**e**) Go/No-Go reaction time in milliseconds.

Baseline-to-week-10 Digit Span Forward results indicated no significant improvements from pre- (*M* = 6.2; *SD* = 1.87) to post-intervention (*M* = 6.8; *SD* = 1.75) [*t*(9) = −1.5, *p* = 0.17], with a small effect size (Cohen’s *d* = 0.13) (see Figure 4a). Digit Span Backward results did not meet the assumption of normality, as assessed via the Shapiro–Wilk test. There were no significant differences on the Digit Span Backward results pre- (median = 5) and post-intervention (median = 5), with *V* = 16, *z* = −0.26, *p* = 0.79, *r* = −0.08 (see Figure 4a). The Trails Making Test A indicated no significant difference between pre- (*M* = 0.85 min, *SD* = 0.26) and post-intervention (*M* = 0.91 min, *SD* = 0.31) [*t*(9) = −0.65, *p* = 0.53] (see Figure 4b). The Cohen’s *d* for Trail Making Test A was 0.22, indicating a small effect size. Similarly, the Trail Making Test B showed no significant difference between pre- (*M* = 1.25 min, *SD* = 0.42) and post-intervention (*M* = 1.29 min, *SD* = 0.50) [*t*(9) = −0.20, *p* = 0.84], with an effect size of d = −0.08. An outlier in the Trails B test, evidenced by a maximum value of 2.48 min, contributed to positive skewness (1.72) and elevated kurtosis (3.23). Without the outlier, the paired *t* test remained insignificant [*t*(8) = 0.61, *p* = 0.56]; however, the Cohen’s *d* increased to −0.25, indicating a small effect size.

Stroop Task interference mean reaction time ratio for pre- and post-testing was calculated by subtracting incongruent and congruent individual participants’ mean correct response reaction time and dividing that difference by the individual participants’ mean correct congruent response reaction time [20]. Paired samples *t* test indicated no significant improvements between pre- (*M* = 0.34, *SD* = 0.24) and post-intervention (*M* = 0.27, *SD* = 0.29) [*t*(9) = 0.99, *p* = 0.35] (see Figure 4c). Cohen’s *d* for Stroop Task interference ratio was −0.28, indicating a small effect size.

The Go/No-go task was analyzed with Wilcoxon signed-rank test for five participants as data were not available for the remaining participants due to software issues. The results indicate no significant difference between pre- and post-intervention for Go trials and No-Go trials (see Figure 4d,e). Go trials revealed non-significant improvements in accuracy from pre- (median = 83.9%) to post-intervention (median = 89.3%), *V* = 3, *z* = −0.55, *p* = 0.58, *r* = −0.24. No meaningful changes were observed in reaction time pre- (median = 162.75ms) and post-intervention (median = 161.46ms), *V* = 6, *z* = −0.24, *p* = 0.81, *r* = −0.12. Similarly, No-Go trials revealed non-significant improvements in accuracy from pre- (median = 90.18%) to post-intervention (median = 94.64%), *V* = 1, *z* = −1.63, *p* = 0.10, *r* = −0.73. No-Go reaction times showed an increase from pre-intervention (median = 190.12 ms) to post-intervention (median = 208.35 ms). However, this change was not statistically significant, with *V* = 7, *z* = 0, *p* = 1, *r* = 0.

### 3.3. Clinical Outcomes

Assumption of normality was not met for the following tests, as assessed by the Shapiro–Wilk test: BSSI and two domains of the Quality-of-Life Scale, namely, personal development and fulfilment; and recreation.

The Wilcoxon signed-rank test was used to determine if there were improvements in BSSI scores. The results indicated a significant difference between pre- (median = 17) and post-intervention (median = 12.5), *V* = 51.5, *z* = −2.40, *p* = 0.02. BSSI results indicate a significant decrease in intensity of suicidal ideation with a large effect size of *r* = −0.76, suggesting the potential role of the intervention lessening suicidal ideation intensity.

QIDS scores were analyzed using a paired samples *t* test, resulting in no significant changes between pre- (*M* = 18.6, *SD* = 6.26) and post-intervention (*M* = 17, *SD* = 6.57) [*t*(9) = 1.16, *p* = 0.28]. Cohen’s *d* for QIDS scores was −0.25, indicating a small effect size.

Quality-of-Life Scale results were analyzed across five domains: material and physical well-being; relationship with other people; social community and civic activities; personal development and fulfilment; and recreation. Significant improvements were observed in material and physical well-being (pre-intervention: *M* = 7.4, *SD* = 2.8; post-intervention: *M* = 8.5, *SD* = 2.22; *t*(9) = −2.19, *p* = 0.05; Cohen’s *d* = 0.44), relationship with other people (pre-intervention: *M* = 15.5, *SD* = 3.72; post-intervention: *M* = 19.2, *SD* = 3.71; *t*(9) = −2.43, *p* = 0.04; Cohen’s *d* = 1.0), and social community and civic activities (pre-intervention: *M* = 7.8, *SD* = 2.04; post-intervention: *M* = 10.1, *SD* = 2.42); *t*(9) = −2.91, *p* = 0.02; Cohen’s *d* = 1.03). No significant differences were found in personal development and fulfilment (pre-intervention: median = 16.5; post-intervention: median = 18.5, *V* = 13.5, *z* = −1.01, *p* = 0.31, *r* = −0.32) and recreation (pre-intervention: median = 16.5; post-intervention: median = 19.5, *V* = 7.5, *z* = −1.40, *p* = 0.16, *r* = −0.44). These findings suggest that the intervention may contribute to improvements in quality of life, particularly in the domains of material and physical well-being, relationship with other people, and social community and civic activities (see Table 2). However, due to the absence of a control arm, the impact of receiving concurrent psychotherapy cannot be excluded.

### 3.4. Feasibility and Acceptability

Recruitment for this study was conducted over an eight-month period. During that time, 61 people expressed an interest generated from Facebook advertisements (33%), posters in the community (21%), a database of consent to be contacted for future studies (20%), referrals from the research team (15%), and from the laboratory’s website (11%). On average, two participants were enrolled per month over the recruitment period. Among those who started the intervention, the completion rate was 66.7%, while retention rate 55.6%.

**Figure 5:**
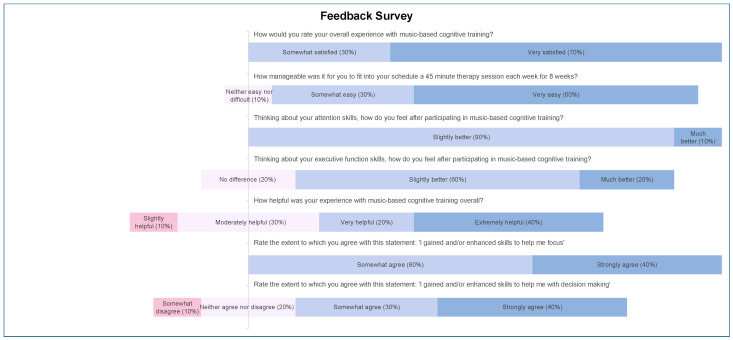
Divergent bar chart of music-based cognitive training feedback survey.

Participant feedback of their experience with NMT and music-based cognitive interventions are illustrated in Figure 5. All participants expressed positive statements towards their experience with music-based cognitive training (ranging from somewhat to very satisfied). When asked how manageable it was to fit the sessions into their schedules, 90% reported that it was easy (somewhat or very easy), and 10% expressed neutral thoughts (neither easy nor difficult). All participants noticed improvements in attention after participating in sessions (slightly or much better). Regarding executive function skills, 80% noticed an improvement (slightly or much better), and 20% reported no difference. Participants generally found the sessions helpful, with 60% finding them very or extremely helpful, 30% rating them moderately helpful, and 10% indicating slight helpfulness.

The QCA of open-ended survey questions yielded three central themes: benefits, challenges, and acceptability of participating in music-based cognitive training sessions. Survey questions are listed in Table 3.

The analysis identified several emerging categories within the themes. Among these findings, the most frequently mentioned categories within the theme of benefits were increased focus and enjoyment (see Table 4). Within the theme of challenges, the most cited aspects were musical challenges and transportation barriers (see Table 5). Regarding acceptability, all participants mentioned a continued interest in NMT (see Table 6).

## 4. Discussion

The experience of cognitive dysfunction in individuals with suicide risk and MDD is a daily challenge, and there is an increasing need to develop effective interventions to support these individuals [21,22]. Our research provides new insights into addressing cognitive function and reducing suicidal ideations using music-based interventions, specifically two neurologic music therapy applications: MACT and MEFT for cognitive function. The MACT and MEFT protocols utilized in this study were derived from the Oxford Handbook of Neurologic Music Therapy [14]. Adaptions were made based on the participant’s progress, which aligns with the protocol’s flexibility. Specifically, the music provided by the NMT was tailored and modified based on the participant’s task completion, preferences, and sensory needs.

Findings from this study reveal that participating in eight sessions of MACT and MEFT led to a significant improvement in suicidal ideation intensity. This reduction highlights the clinical relevance of the intervention in decreasing the intensity of suicidal ideation, suggesting its potential as a valuable therapeutic approach. Participating in music-based interventions engages and alters the orbitofrontal cortex (OFC), anterior cingulate cortex (ACC), amygdala, ventral striatum, insula, hippocampus, and ventromedial prefrontal cortex (vmPFC) [23]. MACT and MEFT have potential to directly target key functions such as reward processing, cognitive integration, and emotional responses. Similarly, the neurobiological mechanisms involved in suicidal ideation include alterations in the OFC, ACC, and amygdala [24]. This overlap suggests that NMT could modulate the brain regions impacted in suicidal risk, making it a potentially complementary adjunct to other interventions.

Psychological mechanisms may also contribute to the decrease in suicidal ideation intensity. Active engagement in music-based cognitive interventions provides a space for different thought patterns, potentially fostering a sense of accomplishment and self-expression. This aligns with existing literature highlighting the therapeutic benefits of music-based interventions in mental health treatment [25,26]. Additionally, social and environmental factors, such as weekly in-person support, may offer immediate relief by changing the environment and reducing feelings of isolation. Consistent social interaction, a supportive environment, and therapeutic relationships play a crucial role in mitigating suicidal thoughts as they provide a sense of community and belonging [27]. These combined mechanisms may contribute to the reduction in suicidal ideation intensity and offer a sense of belonging, underscoring the potential of NMT applications for mental health.

Depressive symptoms, as measured by the QIDS, remained within the severe range from baseline to post-intervention. The absence of significant improvements in depressive symptoms, in contrast to the observed reduction in suicidal ideation intensity, may be attributed to several factors. The music-based intervention was primarily designed to enhance functional aspects of cognition rather than directly target depressive symptoms. This focus on cognitive function may explain the lack of significant change in depressive symptoms and suggests that the intervention may target mechanisms specifically related to suicidal ideation, rather than general depressive symptoms. Additionally, the intervention’s duration of eight sessions may have been insufficient to elicit notable changes in depressive symptoms. Therefore, longer intervention periods should investigate the impacts of extended music-based interventions on depressive symptoms [28]. Previous research using music-based interventions to address depressive symptoms indicates an improvement in depressive symptoms; however, generalizability of the results is difficult, and caution is necessary when interpreting results [28,29,30]. While depressive symptoms encompass a range of emotional and physical experiences, suicidal ideation is a more specific acute onset of psychological distress. These findings highlight that depressive symptoms and suicidal ideations may not always be related, challenging common assumptions and conceptualizations of their relationship [31]. This suggests that more distinct music-based interventions may be needed to address depressive symptoms. However, the reduction in suicidal ideation intensity observed in this study suggests that NMT applications may have a more immediate effect on alleviating acute distress, even if there were no significant impacts on depressive symptoms.

There was also a significant improvement on the Quality-of-Life Scale, specifically in the domains of material and physical wellbeing, relationship with other people, and social community and civic activities. This improvement is particularly relevant for individuals experiencing suicidal ideation, who frequently experience feelings of isolation and hopelessness. An increase in quality of life may help mitigate suicide risk and enhance coping mechanisms, increase motivation for continued therapeutic engagement, and enhance social integration for this clinical population [32]. Previous research has demonstrated that music-based interventions can enhance quality of life and offer a sense of connection and belonging [30,33,34]. The in-person delivery of the intervention may have contributed to enhancing social integration and community, contributing to improvements in quality of life. Exploring the potential of group sessions versus individual sessions could be valuable as group settings might further enhance social integration and community, providing additional support.

The feasibility of this study was demonstrated through several key findings. Recruitment was conducted through a multi-faceted approach reaching different segments of the clinical population. Facebook advertisements was the most successful channel, indicating that social media is a powerful tool. Expanding to other platforms could further increase reach. In combination with community posters, which were the second most effective channel, combining this with digital posters through local online community groups might enhance recruitment. This study had a good retention rate of 55.6%, with ten out of eighteen enrolled participants completing the study. This higher retention rate, compared to what is noted in suicide research, may be attributed to the novelty of music-based interventions, which could have enhanced participation motivation and adherence [35]. Notably, 100% of the participants were satisfied with their experience, and everyone expressed continued interest in NMT sessions.

Participants reported notable improvements in their cognitive abilities, with 100% indicating enhanced attentional skills and 80% noting better executive function skills. This increased self-efficacy in cognitive abilities suggests a promising potential for future growth in these areas, as well as psychological progress and overall confidence [36]. Despite these positive self-reports, standardized assessments did not show significant improvements. Although significance was not reached, raw mean scores showed improvements in auditory attention, short-term memory, mental flexibility and set shifting, interference control, and inhibition. This discrepancy could be due to the lack of established best practice for assessing cognitive performance in individuals with MDD. Current assessments fail to provide a comprehensive picture and may not be sensitive enough to capture incremental changes or improvements [6,37].

It is important to recognize the limitations of this study. As this was a pilot study that was underpowered for statistical tests, effect sizes provide some insight as to what may be achieved in a powered study. The MACT and MEFT applications used in this study were specifically designed to address impaired neurocognitive mechanisms, such as short-term memory, auditory attention, mental flexibility and set-shifting, cognitive control, problem solving, and decision making, through structured tasks that paralleled these functions through music. However, it is important to note that cognitive assessments were evaluated only at baseline and post-intervention, which introduces the potential for confounding variables to influence participant performance. Post-intervention assessments were conducted either on the same day or the following day after the last music-based cognitive training session. This timing could be a limitation regarding the retention of cognitive improvements, as immediate post-intervention assessments may not accurately reflect the long-term cognitive benefits of the NMT interventions. Future studies should consider re-assessment one week later to better understand the lasting effects and to determine if cognitive improvements are sustained over time.

While our findings indicate a significant reduction in suicidal ideation intensity after MACT and MEFT, which are evidence-based standardized interventions [14], it is important to consider the possibility that other factors that may have influenced these results. Suicidal ideation can fluctuate over short periods, and improvements may be partially attributed to such fluctuations [38,39]. Additionally, the therapeutic setting and the interaction with the therapist may have contributed to improvements in suicidal ideation, independent of NMT itself [26].

Due to the small sample size and the inclusion of participants with a stable medication regimen and more than 12 psychotherapy sessions for their current depressive episodes, previous treatments and co-morbidities were not evaluated in the analysis. Future studies with larger sample sizes and more detailed medical histories could further explore the potential impact of these factors.

Additionally, the dosage of the therapy, consisting of only eight sessions of 45 min each, may not have been sufficient to observe significant changes in cognition. While NMT lacks specific guidelines for dosage of cognitive interventions like MACT and MEFT, there are recommendations for applications in the sensorimotor and speech and language domains, such as rhythmic auditory stimulation (RAS), melodic intonation therapy (MIT), and rhythmic speech cueing (RSC), which include specific session durations and frequencies. These recommendations vary depending on specific aims and diagnostics; however, they generally suggest multiple sessions per week (3–5 sessions), lasting between 30 and 60 min, over a period of 3–11 weeks [14,40,41,42]. These guidelines could serve as indicators for MACT and MEFT interventions, suggesting that longer and more frequent sessions might be necessary to produce measurable cognitive improvements. Implementing an at-home program could provide more frequent and sustained engagement, potentially leading to more significant cognitive changes. This approach would allow participants to integrate and transfer applications learned during sessions into their daily routines, thereby enhancing the potential for long-term cognitive benefits.

This study recruited only participants assigned female at birth, which limits the generalisability of the findings to other populations. Although this was not the intention, the recruitment methods used, such as Facebook and community posters, may have inadvertently favored individuals assigned female at birth who are active on social media or engaged in community activities. These limitations should be considered when interpreting the results.

It is crucial to acknowledge the challenges participants faced in attending sessions, including transportation barriers and symptoms of depression that hindered their willingness to leave the house. These obstacles highlight the necessity for flexible intervention delivery methods. An online option may warrant further exploration, as it could enhance accessibility, participation, and retention. Investigating whether similar outcomes for both suicidal ideation and cognitive function can be achieved in an online format would provide valuable insights into the effectiveness of music-based cognitive interventions across diverse settings.

## 5. Conclusions

This pilot intervention study used an innovative approach integrating mental health, neurorehabilitation, and music. Using neurologic music therapy applications (i.e., MACT and MEFT) provided valuable insights into its feasibility and acceptability with individuals with MDD and suicide risk. MACT and MEFT have often been used with other clinical populations (e.g., acquired and traumatic brain injury) and have shown improvements in cognitive function [19,43]. This study demonstrates that these applications can also be successfully implemented for individuals with mental health challenges, specifically depression and suicidal risk. The in-person protocol enabled participants to engage with the music in real time, though it restricted participation to those who could physically access the study site. A robust safety protocol was in place, and no high-risk incidents or adverse events occurred. However, the study’s small sample size and single-arm design limit the broader applicability of the results. Additionally, while the broad inclusion criteria reflected real-world conditions of this clinical population, it also increased variability, making it harder to identify specific outcomes for different subgroups.

Future research should utilize a randomized control paradigm and include a neuroimaging component to understand further the efficacy and underlying neural mechanisms of cognitive training with MACT and MEFT with this clinical population. Additionally, exploring neurodiversity and sensory profiles within this clinical population would yield valuable insights and enable us to explore the broader implications of MACT and MEFT applications. These approaches may uncover specific cognitive and neural patterns that contribute to vulnerability and resilience in affected individuals.

In conclusion, the present pilot study demonstrates that eight individual music-based cognitive sessions completed within a 10-week period is a feasible and acceptable therapy. Furthermore, improvements in self-reported increased confidence in attentional and executive function skills, suicidal ideation intensity, and overall quality of life were observed, therefore supporting the potential of NMT interventions as an effective therapy for individuals with MDD and suicide risk. The substantial reduction in intensity of suicidal ideation is particularly promising, highlighting the intervention’s profound impact, while the initial cognitive improvements, though modest, pave the way for further exploration and enhancement.

## Figures and Tables

**Table 1:** Baseline characteristics of study participants.

Characteristic	Participants (n = 10)	
	Mean	SD
Age, y (range)	41.7 (26–69)	13.57
	N	
Female sex at birth	10	
Gender		
Woman	9	
Non-binary/other ^a^	1	
Ethnicity		
South Asian	2	
Latino	1	
White	7	
Marital status		
Never married	7	
Married/domestic partnership	2	
Divorced	1	
Education		
High school graduate	1	
Some college, no degree	3	
Associate degree	2	
Bachelor’s degree	1	
Master’s degree	3	
Occupation status		
Employed	3	
Unemployed, looking for work	1	
Disabled (permanently/temporarily)	5	
Retired	1	
Lifetime suicide attempt	7	
Number of past suicide attempts ^b^		
0	3	
1	2	
≥2	5	
Comorbidities		
Agoraphobia	3	
Antisocial personality disorder	2	
Binge eating disorder	1	
Borderline personality disorder	4	
Generalised anxiety disorder	3	
Obsessive compulsive disorder	1	
Panic disorder	4	
Post traumatic stress disorder	4	
Social anxiety disorder	1	
Psychotropic medication use at baseline ^c^	9	
Antidepressants ^d^		
SSRI	2	
SNRI	4	
NDRI	2	
SARI	1	
TCA	1	
Antipsychotics	5	
Anti-epileptic	2	
Anxiolytics and hypnotics		
Benzodiazepines	3	
Nonbenzodiazepine hypnotic	1	
Stimulants	2	
Cannabinoid	1	

^a^ Includes genderqueer, gender non-conforming, and neither exclusively man nor woman. ^b^ Number of past suicide attempts in lifetime with no attempts in the last 3 months. ^c^ Stable medication of at least four weeks. ^d^ Antidepressant abbreviations: selective serotonin reuptake inhibitor (SSRI); serotonin-norepinephrine reuptake inhibitor (SNRI); norepinephrine–dopamine reuptake inhibitor (NDRI); serotonin antagonist and reuptake inhibitor (SARI); tricyclic antidepressant (TCA).

**Table 2:** Clinical Outcomes at baseline and post music-based cognitive intervention.

Clinical Outcome	Baseline		Post-Intervention		*p*-Values	Effect Size
	Mean	SD	Mean	SD		
BSSI	19.1	5.84	12.7	7.33	0.02 *	*r* = −0.76
QIDS	18.6	6.26	17	6.57	0.28	*d* = −0.25
Quality of Life Scale						
Material and Physical Well-being	7.4	2.80	8.5	2.22	0.05 *	*d* = 0.44
Relationship with Other People	15.5	3.72	19.2	3.71	0.04 *	*d* = 1.00
Social Community and Civic Activities	7.8	2.04	10.1	2.42	0.017 *	*d* = 1.03
Personal Development and Fulfilment	16.3	5.46	18.5	3.67	0.31	*r* = −0.32
Recreation	16.4	5.52	19.1	3.45	0.61	*r* = −0.44

*: The asterik indicates statistical significance.

**Table 3:** Open-ended survey questions.

Questions		
What was the most beneficial aspect of the study for you?		
What was the most challenging aspect of the study for you?		
Is there anything else you would like to say about your experience with the study?		
If it was available, would you be interested in registering for Neurologic Music Therapy sessions?		

**Table 4:** Theme: benefits of participating in music-based cognitive training sessions.

Category	No.	Example Quotation
Increased focus	5	“I found it helped my focus” “helped me focus better at home” “Getting stronger at keeping my focus” “my focus got better” “I can focus more and put all my attention in the musical session”
Decision making processes	1	“being able to have a space to make choices and decisions”
Non-judgmental and safe environment	3	“to not be graded based on my results” “The actual study was light-hearted and made me feel safe” “I feel happy the people like me”
Therapeutic alliance	2	“Seeing the same person every week who was encouraging me in-person”. “I also actually looked forward to seeing Melissa and doing this activity, which is not that common for me” “Nice people conducting the study”.
Receiving feedback	1	“Feedback about how I improved was useful and helped me listen to music in general in other ways”
Enjoyment	6	“was a nice discipline and fun creatively” “It was good to know that I can still enjoy some things”. “Getting to participate in an enjoyable activity” “For me was so fun and I love this session its works for my brain”. “I knew I would enjoy it once I started!” “I enjoyed it”. “I really enjoyed my time, I found it helpful and fun”
Music related benefits	2	“Gaining a little more confidence in my ability to play music”. “This was an eye opening experience and I could see how rhythm can help the brain/mind process at a higher level” “I got a keyboard and have decided to try to teach myself basic piano, skills, to help distract myself when feeling dark”
Contributions to research	1	“To potentially help others learn more about depression”.
Personal growth	4	“Honestly, using my brain in a different way–getting outside of myself for a period of time”. “This study required participation within my comfort zone, it was nice to see others genuinely interested in learning more about what I experience with depression”. “I felt better after every session” “It quietened the negative voices” “Helping me find voice was the most beneficial”.

**Table 5:** Theme: challenges of participating in music-based cognitive training sessions.

Category	No.	Example Quotation
Self-criticism	1	“I would be hard on myself”
Musical challenges	2	“All rhythm-based tasks were a challenge”
Transportation barriers	2	“Sometimes getting to the appointment, especially if [public transit] was delayed because I would be late (or too early!) and would be stressed” “The study required transit I would normally not take due to anxiety/fear, but pushed me to attend anyway”.
Disclosure of information	1	“Finding the balance between being honest enough for correct information to be gathered while avoiding being committed to a hospital stay”.
Depressive symptoms	1	“Because my health issues and depression can keep me from wanting to leave the house, I have had to cancel a few times”

**Table 6:** Theme: acceptability of music-based cognitive training sessions.

Category	No.	Example Quotation
Integration of flexibility and structure	1	“It was a good mix of structure and creativity”.
Access to free services	2	“It would be great to have this as a free program as well because I found it to be really helpful personally” “Yes, if it was financially feasible”
Continued interest in NMT	10	“Yes, if it was financially feasible” “Absolutely. This setting has been a positive way to learn how to focus more and dissociate less”. “Definitely yes”. “Yes, absolutely” “100 percent I would happily invest time and money into participating”

## Data Availability

The raw data supporting the conclusions of this article will be made available by the authors on request.
